# An In Silico Design of Peptides Targeting the S1/S2 Cleavage Site of the SARS-CoV-2 Spike Protein

**DOI:** 10.3390/v15091930

**Published:** 2023-09-15

**Authors:** Chian Ho, Wan Fahmi Wan Mohamad Nazarie, Ping-Chin Lee

**Affiliations:** 1 Faculty of Science and Natural Resources, Universiti Malaysia Sabah, Kota Kinabalu 88400, Sabah, Malaysia; hochian2015@gmail.com (C.H.); wanfahmi@ums.edu.my (W.F.W.M.N.); 2 Biotechnology Research Institute, Universiti Malaysia Sabah, Kota Kinabalu 88400, Sabah, Malaysia

**Keywords:** SARS-CoV-2, S1/S2 cleavage site, peptides, HADDOCK, molecular dynamics simulation

## Abstract

SARS-CoV-2, responsible for the COVID-19 pandemic, invades host cells via its spike protein, which includes critical binding regions, such as the receptor-binding domain (RBD), the S1/S2 cleavage site, the S2 cleavage site, and heptad-repeat (HR) sections. Peptides targeting the RBD and HR1 inhibit binding to host ACE2 receptors and the formation of the fusion core. Other peptides target proteases, such as TMPRSS2 and cathepsin L, to prevent the cleavage of the S protein. However, research has largely ignored peptides targeting the S1/S2 cleavage site. In this study, bioinformatics was used to investigate the binding of the S1/S2 cleavage site to host proteases, including furin, trypsin, TMPRSS2, matriptase, cathepsin B, and cathepsin L. Peptides targeting the S1/S2 site were designed by identifying binding residues. Peptides were docked to the S1/S2 site using HADDOCK (High-Ambiguity-Driven protein–protein DOCKing). Nine peptides with the lowest HADDOCK scores and strong binding affinities were selected, which was followed by molecular dynamics simulations (MDSs) for further investigation. Among these peptides, BR582 and BR599 stand out. They exhibited relatively high interaction energies with the S protein at −1004.769 ± 21.2 kJ/mol and −1040.334 ± 24.1 kJ/mol, respectively. It is noteworthy that the binding of these peptides to the S protein remained stable during the MDSs. In conclusion, this research highlights the potential of peptides targeting the S1/S2 cleavage site as a means to prevent SARS-CoV-2 from entering cells, and contributes to the development of therapeutic interventions against COVID-19.

## 1. Introduction

The novel *Betacoronavirus*, Severe Acute Respiratory Syndrome Coronavirus 2 (SARS-CoV-2), caused the COVID-19 pandemic disease [1]. It often causes mild-to-severe respiratory symptoms, such as cough, sore throat, and/or fever, with varying degrees of severity at different clinical stages of the disease [2]. SARS-CoV-2 is an enveloped virus that possesses positive-sense RNA [3]. The virus, about 5–200 nm in diameter, contains four structural proteins: the spike protein (S), the envelope protein (E), the membrane protein (M), and the nucleocapsid protein (N) [4]. The S protein is responsible for SARS-CoV-2’s binding and entry to the host cell, causing infection. This protein is also the target for neutralizing antibodies in an infection response. The S protein is trimeric, with each protomer comprising a single polypeptide chain. It possesses the receptor-binding domain (RBD), the S1/S2 and S2 cleavage sites, and the heptad-repeat (HR1 and HR2) regions, which play essential roles in viral entry into host cells [3].

The RBD of the S protein first attaches to the host cell’s angiotensin-converting enzyme 2 (ACE2) receptor for entry into the host cell. After the initial attachment, the S protein is cleaved by host proteases at the S1/S2 and S2 cleavage sites into S1 and S2 subunits. The cleaving of the S protein causes an irreversible conformational change that exposes the fusion peptide, which allows it to be inserted into the host-cell membrane. The three HR2s will then bind to three HR1s to form the fusion core, which brings the viral- and host-cell membranes into proximity and facilitates their fusion, thereby enabling the entry of the viral genome into the host cell [3,5]. Another route of entry is via clathrin-mediated endocytosis. This entry route involves the cleaving of the S protein at the S1/S2 cleavage site by the host proteases cathepsin L and cathepsin B at an acidic pH. Fusion of the viral membrane with endosomes, then allows the release of the viral genome into host-cell cytoplasm [6,7]. This route seemed to be preferred by Omicron BA.1 and BA.2, which showed reduced cleavage activity by the host cell TMPRSS2 and increased cleavage activity by the endosomal proteases [8].

The mutations recorded on several variants of SARS-CoV-2 enhance this infection mechanism. The K417N (Beta, Gamma, Omicron variants)/K417T (Delta variant) mutation, E484K (Beta, Gamma, Delta variants)/E484A (Omicron variant) mutation, N501Y mutation (Alpha, Beta, Gamma, and Omicron variants), and L452R mutation (Delta variant) recorded at the RBD region all caused an increased binding affinity of the S protein to the ACE2 receptor [9]. Apart from this, the D614G mutation near the S1/S2 cleavage site of all Alpha, Beta, Gamma, Delta, and Omicron variants enhanced spike cleavage and the infectivity of the virus. In addition, the H655Y mutation found in the Gamma and Omicron variants and the N679K mutation near the S1/S2 cleavage site can increase the rate of spike cleavage. Other important mutations at the S1/S2 cleavage site are P681R for the Delta variant and P681H for the Alpha variant and the Omicron variant, which enhanced cleavage at the S1/S2 cleavage site [9,10].

The U.S. Food and Drug Administration (FDA) has approved three drugs for treating COVID-19: an antiviral drug and two immune modulators [11]. The antiviral drug Veklury (remdesivir) targets the RNA-dependentRNA polymerase (RdRp) of SARS-CoV-2 and inhibits viral RNA transcription [12]. Actemra (tocilizumab), an interleukin-6-receptor-blocking immune modulator, and Olumiant (baricitinib), a Janus Kinase (JAK) 1 and 2 inhibitor, are also approved by the FDA for the treatment of COVID-19 [11,13]. The FDA has granted Emergency Use Authorization (EUA) to several other antiviral drugs and immune modulators to combat COVID-19. However, due to the circulating SARS-CoV-2 variants, the FDA has revoked all previously EUA-approved monoclonal antibodies for COVID-19 treatment. The mutated variants have developed resistance towards monoclonal antibodies, such as REGEN-COV, bamlanivimab, etesevimab, bebtelovimab, and sotrovimab, resulting in ineffective treatment [11,14,15,16,17].

It has been observed that the number of FDA-approved drugs for COVID-19 treatment is few. Furthermore, no antiviral drugs inhibit the entry of SARS-CoV-2 into host cells. In addition, none of the FDA-approved or FDA–EUA-approved drugs are peptide drugs. Hence, developing a peptide drug that can inhibit SARS-CoV-2 viral entry is of great interest.

Novel synthetic peptides are considered to have great potential as therapeutic drugs. Peptides are short-chained amino acids that can be designed and synthesized via chemical or molecular biological techniques [18]. They are small and can easily enter into cells to bind to the active domains of targeted molecules. Peptides also exhibit a high potency of action and high target specificity. They have high selectivity amid a wide range of targets. They can be metabolized into amino acids, are low in toxicity, and have fewer side effects. Therapeutic peptides can elicit various chemical and biological reactions [19]. Peptides have a lower manufacturing cost than protein-based medications [20]. Thus, synthetic peptides offer much potential as a COVID-19 medication. There were studies on peptides designed to target the RBD and HR1 regions of the S protein, however, less information is available on peptides designed to target the S1/S2 cleavage site. There are also peptides designed to target the ACE2 receptor and host proteases such as TMPRSS2, cathepsin L, and furin [21]. The S protein’s S1/S2 cleavage site could be a potential area for research on novel COVID-19 drugs [22]. Thus, this study aimed to create an in silico design of peptides that target the S protein’s S1/S2 cleavage site to prevent viral entry or infection.

Furin, trypsin, cathepsin B, cathepsin L, matriptase, and TMPRSS2 are some of the host proteases involved in SARS-CoV-2 infection [23]. These proteases possess the catalytic triad that can cleave the S protein at the S1/S2 cleavage site. A catalytic triad comprises an acidic residue (often aspartic acid, D, or asparagine, N), a basic residue (usually Histidine, H), and a nucleophile. It breaks peptide bonds. The acid residue polarises the basic residue first, and then the basic residue activates the nucleophile. The nucleophile then attacks the substrate’s carbonyl carbon, generating a tetrahedral intermediate, followed by the release of the first product. The presence of water molecules causes the formation of a new tetrahedral intermediate, followed by the release of the second product and the regeneration of free protease [24]. Hence, the host proteases’ catalytic triad and the S protein’s S1/S2 cleavage site are the key residues involved during cleavage by host proteases. The catalytic triad of furin is D153, H194, and S368; trypsin, H63, D107, and S200; matriptase, H656, D711, S805; cathepsin B, C108, H278, N298; cathepsin L, C138, H276, N300; and TMPRSS2, H296, D345, S441 [25]. The active residues for the S1/S2 cleavage site are Y674–I693, where the peptide bond between R685 and S686 is cleaved. 

Bioinformatics tools enable the prediction or study of the interaction between a large number of molecules, such as protein peptides and protein ligands. This study applied bioinformatics tools for peptide design and screening potential peptide inhibitors that can bind to the S protein’s S1/S2 cleavage site. Structural analysis was conducted on the S protein-host cell proteases complex to search for key residues. Peptides targeting the S protein were then designed. Physicochemical and absorption, distribution, metabolism, excretion, and toxicity (ADMET) analysis on potential peptides provide preliminary insight into the pharmacokinetics of the peptides. Molecular dynamics simulation (MDS) analyses enable the determination of the compactness and stability of the protein–peptide complex and quantify the interaction strength of the S protein with the designed peptides.

## 2. Materials and Methods

### 2.1. Data Mining and Homology Modelling

The FASTA sequence of SARS-CoV-2 S protein (PDB ID: 6VXX, from the wildtype Wuhan-Hu-1 strain) was obtained from the RCSB Protein Data Bank [26,27]. Full-length S protein was generated using SWISS-MODEL [28]. The structure 6XR8.1.A was chosen as the template for S protein with its sequence identity at 100%, GMQE (Global Model Quality Estimate) score of 0.87, and QMEAN (Qualitative Model Energy ANalysis) score of −1.26.

Host cell proteases selected for this study were furin, trypsin, TMPRSS2, matriptase, cathepsin B, and cathepsin L [23,29,30]. As no reference structure of TMPRSS2 was available in the RCSB Protein Data Bank, the FASTA sequence of TMPRSS2 (O15393) was retrieved from UniProt [25]. The catalytic domain of TMPRSS2 was generated using SWISS-MODEL [28]. The structure 1Z8G.1.A was chosen as the template for TMPRSS2 with its sequence identity at 33.62%, GMQE score of 0.48, and QMEAN score of −1.81. The model was saved as a PDB file.

PDB structures of furin (PDB ID: 4OMC), trypsin (PDB ID: 2RA3), matriptase (PDB ID: 4IS5), cathepsin B (PDB ID: 6AY2), and cathepsin L (PDB ID: 2XU3) were obtained from RCSB Protein Data Bank [25,31,32,33,34,35]. 

Before docking, all structures were rid of water molecules and ligands and refined by ModRefiner [36]. 

### 2.2. Molecular Docking and Structural Analysis

HADDOCK [37] was used to dock host proteases with S protein, with the catalytic triad of host proteases and the S1/S2 cleavage site as active residues. Structural analysis on these docked complex structures was performed using PyMOL [38] and UCSF Chimera [39]. The complex structures’ hydrogen bonds and non-polar contacts were studied and recorded. Key amino acid residues involved in the binding interfaces of the docked complex structures were determined. 

### 2.3. Peptides Design

Based on the structural analysis results, key amino acid residues involved in the binding interfaces were used to design peptides that bind to the S1/S2 cleavage site. A total of 1102 peptides with different amino acid compositions and lengths were designed and generated to target the S1/S2 site using PEP-FOLD3 [40]. All peptides were refined with 3Drefine [41] before docking with the S1/S2 site of S protein using HADDOCK [37]. 

PRODIGY [42] was utilized to estimate the peptides’ binding affinity. We picked peptides with the lowest HADDOCK scores, RMSD, and binding affinity. 

### 2.4. Physicochemical and Absorption, Distribution, Metabolism, Excretion, and Toxicity (ADMET) Analyses

The toxicity of the peptides was first analysed using the ToxinPred [43] server. The physicochemical properties of the peptides were analysed using the ProtParam tool on the ExPASy web server [44]. The peptide sequences were first converted into SMILES format using the PepSMI tool to perform ADMET analysis [45]. The ADMET properties of the peptides were then analysed using the pkCSM tool on a web server [46]. 

### 2.5. Molecular Dynamic Simulation

The root mean square deviation (RMSD), root mean square fluctuation (RMSF), radius of gyration (Rg), number of hydrogen bonds with time, and interaction energy of the protein–peptide complex were investigated using the CHARMM36 force field [47] and GROMACS version 2020.1 [48,49]. First, the complex was placed in a simulation box. The system was then solvated and ionized, resulting in a net charge of 0 in the simulated environment. The complex’s energy was then minimized, followed by system equilibration in temperature of 300K and 1 bar pressure before the actual simulation. After that, the simulation was run for 100 nanoseconds. Principal component analysis (PCA) was carried out using GROMACS to analyse essential protein dynamics and to determine whether convergence occurred with a cosine content calculation on the first principal component [48,49].

### 2.6. Analysis with The Omicron Variant S Protein

Peptides showing the best results with the wildtype SARS-CoV-2 S protein were also analysed with the S protein of the Omicron variant, the present Variant of Concern [50]. The FASTA sequence of the Omicron S protein (PDB ID: 7XCO, omicron variant) was obtained from the RCSB Protein Data Bank [27,51]. Full-length Omicron S protein was generated using SWISS-MODEL [28]. The structure 7QTI.1.L was chosen as the template for Omicron S protein with its sequence identity at 99.26%, GMQE (Global Model Quality Estimate) score of 0.73, and QMEANDisCo (Qualitative Model Energy ANalysis DIStance COnstraint) global score of 0.73.

## 3. Results and Discussion

### 3.1. Homology Modelling of S Protein Chain A and TMPRSS2

SWISS-MODEL [28] was used to generate a full-length S protein as the protein structure in the Protein Data Bank lacks the S1/S2 cleavage sites, which is essential in this study. The best model for the generated S protein was chosen from the SWISS-MODEL, as indicated by the GMQE score (0.87) and QMEAN score (−1.26). A higher GMQE score indicates higher reliability of the model built [52]. A QMEAN score around zero indicates good agreement between the model created and the experimental structures of the molecule with equivalent size [53]. 

This study focused on only chain A of the S protein for downstream analysis. Additionally, docking analysis results and molecular dynamics simulation (MDS) analysis on any one chain of the S protein would be similar. In an MDS study on monomeric and dimeric forms of human prion protein carried out by Sekijima et al. (2003), they remarked that the dynamics of both monomer and dimer were similar at different temperatures (300 K and 500 K) as well as in an acidic environment [54]. Very often, MDS on spike protein was performed on its monomeric form. Kalathiya et al. (2020) performed MDS on the monomeric and trimeric forms of the spike protein and used the monomeric form, the functional unit of the spike protein, as a control of the trimer [55]. Sixto-López et al. (2021) performed MDS on wild-type and mutant spike protein monomer for structural insights, while Deganutti et al. (2021) performed MDS on the monomer of spike protein RBD-ACE2 complex, as well as the interaction of the spike RBD monomer and ACE2 in the presence of several small molecules [56,57]. With the awareness that the interaction of the peptides might have slight differences due to the structural conformation of the monomeric form and the trimeric form, it was decided that the in silico analysis should first be performed on S protein chain A as a controlled study. MDS should also be carried out on the peptides and the trimeric S protein to study its interaction and binding properties with the trimeric S protein in future.

PROCHECK [58] and ProSA [59] further validated the chosen model. The PROCHECK program is one of the main structure validation tools. The PROCHECK program analyses the geometry of a protein model and compares them with the values of protein structures at the Protein Data Bank (PDB) obtained at a high resolution. Apart from the geometry analysis, PROCHECK also analyses the planarity of the peptide bonds, the energies of hydrogen bonds, and the deviation from expected values of the side chain χ torsion angles. PROCHECK also analyses whether there were bad non-bonded interactions and geometry distortions around the Cα atoms. The program highlights parts of structures with significant deviations from the normal values [60,61]. It was noted that some outliers might be outliers and were not necessarily errors, especially when the protein structure is modelled according to an existing model in the Protein Data Bank. However, if many oddities were found throughout the model, this could indicate that the model is problematic [61]. 

ProSA uses the potential mean force of the Cα atoms in the protein model to evaluate model accuracy. ProSA calculates the z-score and residue energies from these energies and displays the results in two plots. The z-score plot displayed the score for the protein model among the scores of all experimentally determined (X-ray and NMR, distinguished by different colours) protein structures available in the Protein Data Bank. This enables comparison of the overall model quality, and simultaneously enables measuring the deviation of total energy of the protein model. Z-scores that lie outside the range of scores typical for proteins of similar size might indicate problematic structures. The second residue energy plot also determines the protein model quality. A positive energy value indicates that the particular part of the model might be problematic [59].

**Figure 1:**
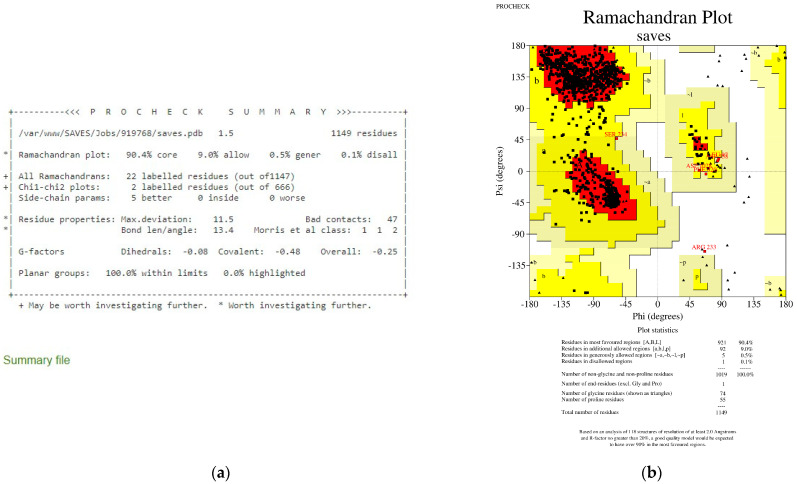
PROCHECK analysis for the S protein chain A shows 90.4% residues in the most favoured regions and an overall G-factor of −0.25. (**a**): PROCHECK summary. (**b**): Ramachandran Plot showing residues in the most favoured region (red); additional allowed regions (yellow); generously allowed regions (light yellow); and disallowed regions (white).

PROCHECK [58] and ProSA [59] confirmed that the homologically modelled S protein chain A had excellent model quality and could be utilized for future investigations. Figure 1 shows the PROCHECK summary of 90.4% of the modelled S protein chain A, and total residues were found in the most favoured regions with the overall G-factor at −0.25. A good quality model should have over 90% in the most preferred regions based on the examination of 118 structures with a resolution of at least 2.0 Angstroms and an R-factor of no more than 20% [58] The overall G-factor is a number that represents the stereochemical characteristic of a protein [62]. Figure 1 also indicates that the S protein chain A is an excellent model since the G-factor is −0.25, larger than −0.5. 

**Figure 2:**
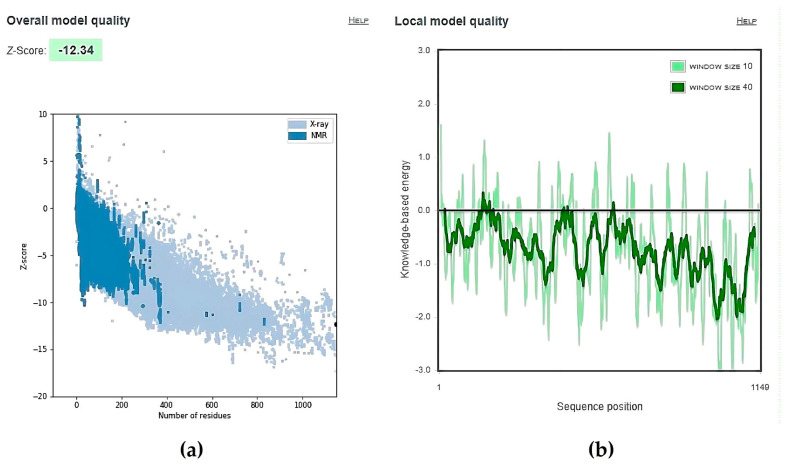
ProSA analysis for the S protein chain A. (**a**): Z-score is −12.34. (**b**): Local model quality where most residues showed negative energy.

Figure 2 shows the ProSA result for the S protein chain A. Figure 2a depicts the z-scores of various proteins in their natural conformations as determined by X-ray crystallography (light blue) or NMR spectroscopy (dark blue) as a function of their length. Most of the scores displayed are for proteins with less than 1000 residues. The z-score of S protein chain A, which has 1149 residues, was among those with natural conformations. Most S protein chain A residues have negative energy, suggesting strong model quality, as shown in Figure 2b. Negative energy shows high-model quality, whereas positive energy indicates a fault or inaccuracy with the construction section [59].

As no structures of TMPRSS2 were available in the RCSB Protein Data Bank, the FASTA sequence of TMPRSS2 (O15393) was retrieved from UniProt [25]. The scavenger receptor cysteine-rich (SRCR) domain and the catalytic domain of TMPRSS2 (residues 144–491) were generated using SWISS-MODEL [28,63]. The best model for TMPRSS2 was chosen, as indicated by its QSQE at 0.48 and QMEAN score at −1.81, showing higher reliability and good agreement between the model built and the experimental structures of the molecule with equivalent size [52,53]. The TMPRSS2 model was further analysed with PROCHECK [58] and ProSA [59] for validation. The homologically modelled TMPRSS2 has high-model quality and may be utilized for further investigation.

**Figure 3:**
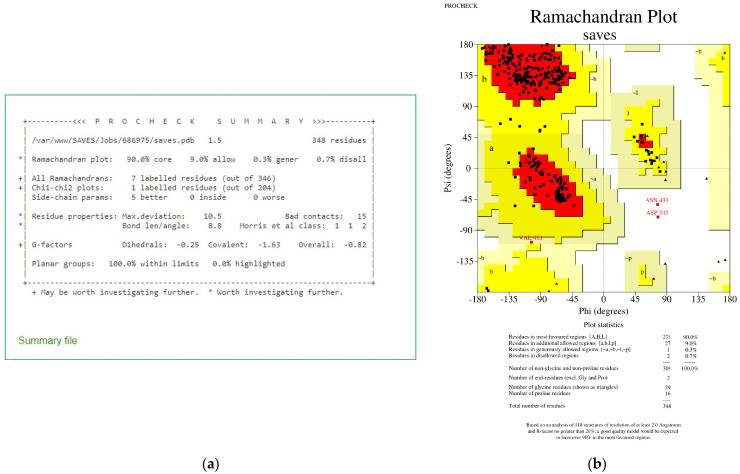
PROCHECK analysis for TMPRSS2 shows 90.0% residues in the most favoured regions and an overall G-factor of −0.82. (**a**): PROCHECK summary. (**b**): Ramachandran Plot showing residues in the most favoured region (red); additional allowed regions (yellow); generously allowed regions (light yellow); disallowed regions (white).

**Figure 4:**
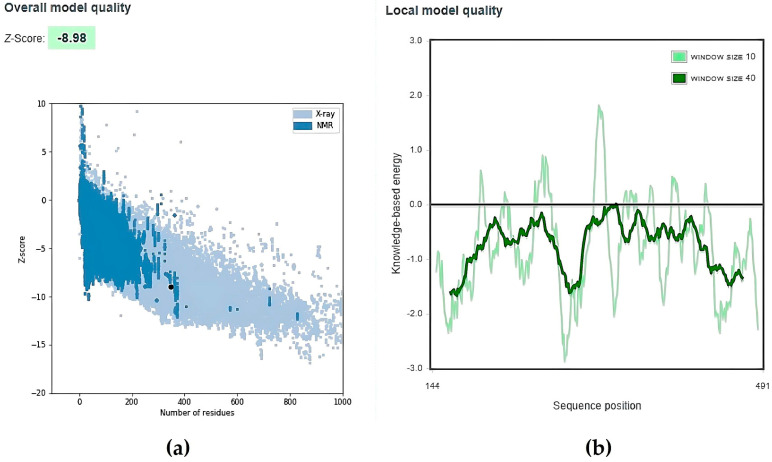
ProSA analysis for TMPRSS2. (**a**): Z-score is −8.98. (**b**): Local model quality where most residues showed negative energy.

The PROCHECK analysis revealed that 90.0% of total residues were found in the most favoured regions with a G-factor of −0.82 overall (Figure 3). The negative value of the G-factor is affected by residues (most are glycine residues) in the disallowed regions of the Ramachandran Plot [64]. Glycine residues have a hydrogen atom instead of a side chain or the C^β^ atom. Hence, it has a wider range of phi and psi angles, and often appears in the disallowed region [65]. Therefore, the overall value of −0.82 is acceptable, indicating that the model is reasonably good, while a value of −1.0 suggests a highly rare stereochemical characteristic [58,66]. Figure 4a revealed that TMPRSS2 has a z-score of −8.98, which is within the range of z-score values associated with native conformations, as demonstrated by other proteins in their native conformations by X-ray crystallography (light blue) or NMR spectroscopy (dark blue) for their length. Figure 4b shows that most of the TMPRSS2 residues have negative energy, indicating that the model is of excellent quality [59].

Before docking, all structures, including the S protein and host cell proteases, were modified to remove steric conflicts and improper geometry and ensure they were in their near-native conformation. The confirmation of the structures will impact docking outcomes. Therefore, refinement is required [67]. Large molecules, such as the S protein and the host cell proteases, may be refined using ModRefiner [36], whereas smaller molecules, such as designer peptides, can be refined with 3Drefine [41].

### 3.2. Molecular Docking and Peptides Design

This study chose HADDOCK for molecular docking because of its strong performance in predicting interaction (CAPRI) events. It also allows for experimental and bioinformatics data, such as active sites or residues, during docking. In contrast, solvent-accessible neighbours are referred to as passive residues and are automatically selected within a 6.5 Å radius of the active residues [68]. 

According to HADDOCK, the best cluster has the lowest HADDOCK score (binding energy), generally the top cluster, with less than 2.0 backbone RMSD [69]. HADDOCK additionally shows the HADDOCK score versus percentage of common contact (FCC), HADDOCK score vs. interface RMSD (i-RMSD), desolvation energy, van der Waals energy, electrostatics energy, and constraints energy vs. i-RMSD for a docking outcome. The HADDOCK score versus FCC plot and the HADDOCK score versus i-RMSD plot were used to examine the models created by docking. The models were satisfactory with i-RMSD less than 4.0 and a fraction of native contacts of 0.1, i-RMSD 2 and a fraction of native contacts of 0.3 being good model predictions, and i-RMSD 1 and a fraction of native contacts of roughly 0.5 being high-quality outcomes [68]. In this study, docking results were analysed based on the above criteria All results that did not meet the criteria mentioned were discarded. 

Table 1 shows the findings of docking the S1/S2 location with host proteases. The results obtained were of good quality, as they were from the top cluster (position 1.0) with the lowest HADDOCK score, RMSD less than 2.0, lowest i-RMSD range of 0.0–1.0 and highest i-RMSD range of 0.0–3.0, and lowest FCC range of 0.3–1.0 and highest FCC range of 0.5–1.0. Pymol [38] and UCSF Chimera [39] were used to perform structural analysis of the docked protein–protein complexes. Table 2 shows the amino acid residues of host proteases that might bind to the S1/S2 cleavage site. These amino acid residues were used to design peptides targeting the S protein’s S1/S2 cleavage site. However, because the S protein S1/S2 cleavage site does not have a fixed structure when it binds to the host proteases, the designed peptides might be in the form of strands, alpha-helixes, or beta-sheets. According to further tests, peptides having an alpha-helix structure have a more stable conformation. As a result, alpha-helix conformation peptides were chosen.

A total of 1102 peptides were designed to target the S protein’s S1/S2 cleavage region using PEP-FOLD3. PEP-FOLD3 allows the structure of a given amino acid sequence to be predicted and peptide models to be generated in PDB format. For every 200 peptide models developed, PEP-FOLD3 generates a set of scores such as sOPEP (Optimized Potential for Efficient structure Prediction), GDT (Global Distance Test), and TM (Template Modelling) for a certain amino acid sequence [40]. The sOPEP score measures the energy of the predicted models, while the similarity of the models is measured by the GDT score [70,71]. Proteins and peptides usually fold into their natural conformations, corresponding to their lowest energy states. Hence, peptides with a low sOPEP score are better. GDT assesses a huge number of superpositions and produces reliable and consistent findings [72]. A GDT value near or equal to 1 suggests that the models are highly similar [73].

This study plotted the sOPEP score with the GDT score to choose the peptide with the lowest energy (near-native conformation) and the highest GDT score (higher similarity). Peptides chosen to dock with the S1/S2 cleavage site must meet specific requirements, such as more than 50% of the 200 models created for a particular peptide sequence being comparable models with RMSDs ranging from 0 to 2. Aside from that, on a displayed sOPEP versus GDT graph, the model must have the lowest sOPEP score and the greatest GDT score. Peptides that did not match the following criteria were excluded from this study.

Docking with the S1/S2 cleavage site further filtered peptides with alpha-helix form, lowest sOPEP score, and highest GDT score. The HADDOCK scores obtained after substituting the residues that can bind to the S1/S2 cleavage site one by one were compared. If a higher HADDOCK score was attained, the peptides were chosen. During docking, the S1/S2 cleavage site residues, Y674–I693, and all residues of peptides targeting the S1/S2 cleavage site were chosen as active residues. As indicated in Table 3, 9 of the 1102 peptides were selected because they were observed to be of good quality. They belong to the top cluster (position 1.0) and have the lowest HADDOCK score, RMSD less than 1.0, lowest i-RMSD range of 0.0–1.1 and highest i-RMSD range of 0.0–3.8, and lowest FCC range of 0.3–1.0 and highest FCC range of 0.5–1.0.

According to the structural study, all peptides interacted with S protein on the S1/S2 cleavage site, i.e., R672 and S673, and can bind with S protein around the S protein–peptide complexes’ S1/S2 cleavage site. Hence, the peptides might inhibit host proteases from attaching to the S1/S2 site, preventing the S protein from activating proteolytically. Table 4 shows the nine peptides’ interaction residues with the S protein’s S1/S2 cleavage site. H denotes hydrogen bond interaction between the residues, while v denotes van der Waals interaction. It was observed that the S protein residues were able to interact with several peptide residues, similar to their interaction with host proteases.

### 3.3. Physicochemical and Absorption, Distribution, Metabolism, Excretion, and Toxicity (ADMET) Analyses

From the physicochemical and ADMET analyses, it can be determined that all peptides are hydrophilic, as revealed by the low % of hydrophobicity and a negative GRAVY score [74]. The theoretical pH of these peptides was between 4.0–4.8, indicating that these peptides are acidic. Hence, the peptides can still be functional in an acidic environment, even within the endosomes (pH 5.5–6.5) or lysosomes (pH 4.5–5.0) [75,76]. Although, it is unstable, with an instability index over 40, and has a half-life of about 1.0 and 1.1 h in a human [44]. Previous studies suggested that proteins or peptides with serine residues at the N-terminal might have a relatively longer half-life in vivo [77]. Hence, if a longer half-life is desired from the peptides, a serine residue can be added at the N-terminal. ADMET analysis showed that the peptides have low skin permeability and are neither a p-glycoprotein substrate nor p-glycoprotein inhibitors. The peptides cannot enter the brain and cannot penetrate the central nervous system (CNS) with log BB < −1 and log PS < −3, which suits the purpose of the peptides as they are not designed to target the brain as well as the CNS. Cytochrome P 3A4 could metabolize the peptides, but they are not cytochrome P inhibitors. In terms of toxicity, the peptides are not carcinogenic or mutagenic, as indicated by the AMES test. They do not cause hepatoxicity as well as skin sensitization. They exhibited a low maximum recommended tolerated dose (MRTD) as all peptides have an MRTD value of less than 0.477 log mg/kg/day. Table 5 shows the result of the physicochemical and ADMET analyses [78]. Hence, the peptides have the potential to be developed into useful drugs to target COVID-19.

### 3.4. Molecular Dynamics Simulation

Molecular dynamics simulation uses Newton’s second law of motion to forecast the velocity and location of atoms or particles over time [79]. The compactness, stability, interaction strength, and S protein docked with the nine peptides were investigated using molecular dynamics modelling. The radius of gyration (Rg) measures the protein–peptide complex’s compactness. An unfolded protein–peptide complex has a variable Rg value, a relatively constant value of Rg indicates that the complex is compact. The stability of the protein–peptide combination is measured by the root mean square deviation (RMSD). Smaller variances indicate a more stable, complicated structure [80]. The root mean square fluctuation (RMSF) evaluates the flexibility or activity of a residue (results not shown). The number of hydrogen bonds between the S protein and the peptides was measured over time. The interaction energy measured the intensity of the interaction between the S protein and the peptides.

PCA is useful for characterizing essential S protein and peptide complex trajectories in a molecular dynamics simulation [81]. PCA analysis generated a series of eigenvectors, each with a respective eigenvalue. In brief, the eigenvector describes a particular motion of the protein–peptide complex and is referred to as a principal component. The eigenvalue of an eigenvector represents the proportion of the motion characterized by the eigenvector (PC) [82]. The cosine content of the first principal component can serve as a negative indicator to determine whether the duration of molecular dynamics simulation is enough for the convergence of the system, and that reliable data can be obtained for further analysis. Cosine content ranged from 0 (no cosine) to 1 (perfect cosine) [83]. A cosine content value close to 1 indicates that the simulation has not converged, while no statement can be made with a value below 0.7 [84,85]. Table 6 shows the S protein and peptide complexes’ PCA and cosine content analysis results. It is observed that the major motion of the protein–peptide complexes (more than 90%) lies in the first few PCs. The cosine content of the S protein and BR606, S protein and BR614 complexes are above 0.7, suggesting that the MDS for these two complexes might need a longer duration. Since all cosine content of the remaining seven complexes was below 0.7, we refrained from analysing the MDS results of the S protein-BR606 and the S protein-BR614 complexes while concentrating on the remaining seven complexes.

**Figure 5:**
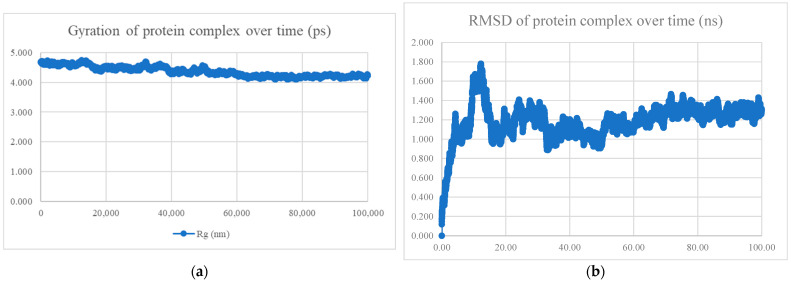
MDS analysis of the S protein and BR556 complex. (**a**) The gyration of the protein complex. A relatively constant line with an overall fluctuation of less than 1 nm shows that the protein complex is compact over time. (**b**) The RMSD of the protein complex. The RMSD initially increased due to minimization and reached stability around 1.2 nm, showing that the protein complex is stable over time. The protein complex’s interaction energy (IE) is −698.794 ± 22.5 kJ/mol, and the mean number of total hydrogen bonds between the protein complex is 10.78.

**Figure 6:**
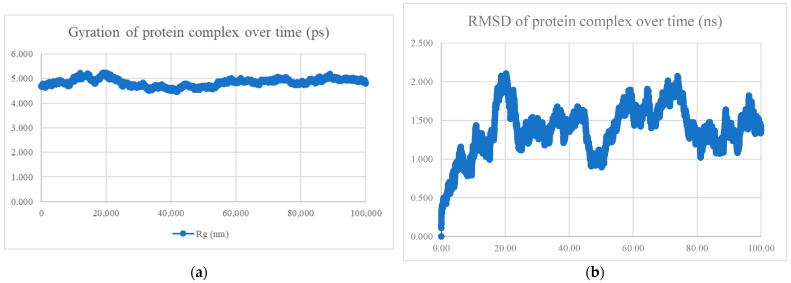
MDS analysis of the S protein and BR563 complex. (**a**) The gyration of the protein complex. A relatively constant line with an overall fluctuation of less than 1 nm shows that the protein complex is compact over time. (**b**) The RMSD of the protein complex. The RMSD initially increased due to minimization and reached stability around 1.5 nm, showing that the protein complex is stable over time. The protein complex’s interaction energy (IE) is −603.423 ± 44.9 kJ/mol, and the mean number of total hydrogen bonds between the protein complex is 7.78.

**Figure 7:**
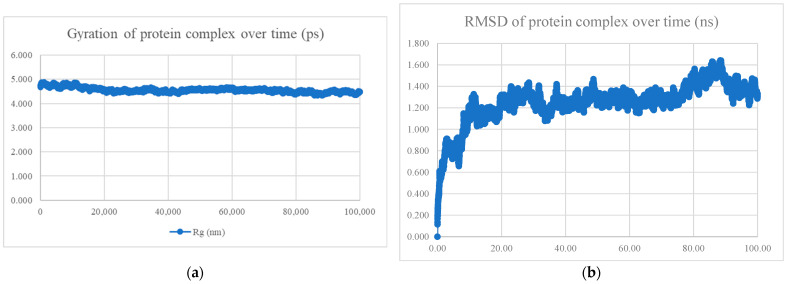
MDS analysis of the S protein and BR573 complex. (**a**) The gyration of the protein complex. A relatively constant line with an overall fluctuation of less than 1 nm shows that the protein complex is compact over time. (**b**) The RMSD of the protein complex. The RMSD initially increased due to minimization and reached stability around 1.3 nm, showing that the protein complex is stable over time. The protein complex’s interaction energy (IE) was −630.395 ± 28.2 kJ/mol, and the mean number of total hydrogen bonds between the protein complex was 6.90.

**Figure 8:**
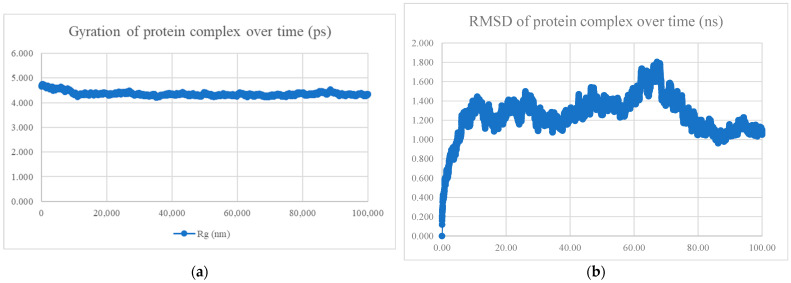
MDS analysis of the S protein and BR582 complex. (**a**) The gyration of the protein complex. A relatively constant line with an overall fluctuation of less than 1 nm shows that the protein complex is compact over time. (**b**) The RMSD of the protein complex. The RMSD initially increased due to minimization and reached stability around 1.1 nm, showing that the protein complex is stable over time. The protein complex’s interaction energy (IE) is −1004.769 ± 21.2 kJ/mol, and the mean number of total hydrogen bonds between the protein complex is 13.85.

**Figure 9:**
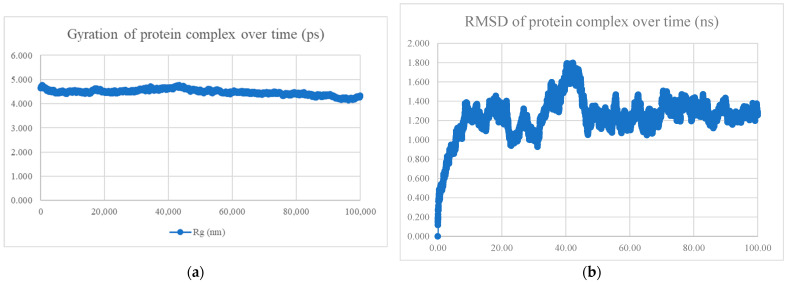
MDS analysis of the S protein and BR599 complex. (**a**) The gyration of the protein complex. A relatively constant line with an overall fluctuation of less than 1 nm shows that the protein complex is compact over time. (**b**) The RMSD of the protein complex. The RMSD initially increased due to minimization and reached stability around 1.2 nm, showing that the protein complex is stable over time. The protein complex’s interaction energy (IE) was −1040.334 ± 24.1 kJ/mol, and the mean number of total hydrogen bonds between the protein complex was 14.51.

**Figure 10:**
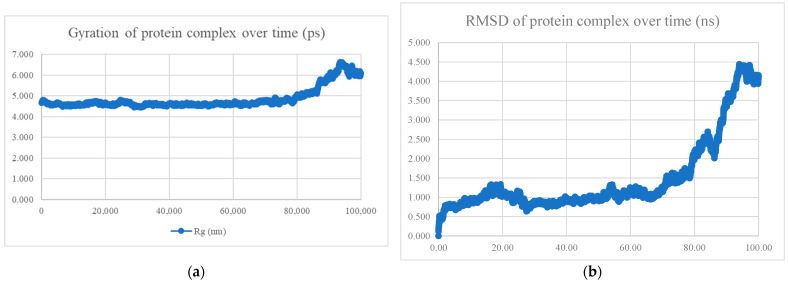
MDS analysis of the S protein and BR605 complex. (**a**) The gyration of the protein complex. (**b**) The RMSD of the protein complex. An increase in gyration and RMSD of the protein towards the end of the molecular dynamics simulation showed that conformational change has occurred. Hence, the protein complex is neither compact nor stable over time. The protein complex’s interaction energy (IE) is −809.339 ± 42.3 kJ/mol, and the mean number of total hydrogen bonds between the protein complex is 11.53.

**Figure 11:**
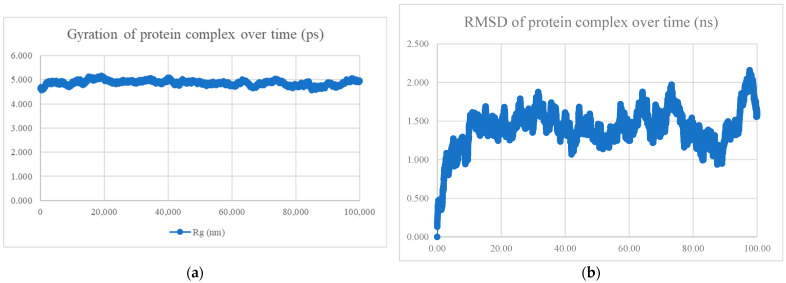
MDS analysis of the S protein and BR610 complex. (**a**) The gyration of the protein complex. A relatively constant line with an overall fluctuation of less than 1 nm shows that the protein complex is compact over time. (**b**) The RMSD of the protein complex. The RMSD initially increased due to minimization and reached stability around 1.5 nm, showing that the protein complex is stable over time. The protein complex’s interaction energy (IE) is −651.934 ± 37.8 kJ/mol, and the mean number of total hydrogen bonds between the protein complex is 7.28.

The MDS findings for the protein–peptide complex are shown in Figure 5, Figure 6, Figure 7, Figure 8, Figure 9, Figure 10 and Figure 11. Apart from the S protein and BR605 complex, the result of Rg and RMSD demonstrated that the complexes were compact and stable over time. There was an initial increase of RMSD due to the complexes being energy minimized before the simulation. The RMSD stabilized with deviations <1.0 nm, showing that the complex is stable over time.

The S protein and peptides BR582 and BR599 had lower interaction energy, −1004.769 ± 21.2 kJ/mol and −1040.334 ± 24.1 kJ/mol, respectively, compared with the other protein–peptide complexes. The protein–peptide complexes with the lowest interaction energy had a higher mean number of hydrogen bonds, 13.85 and 14.51, respectively, formed between them over time. When PRODIGY [30] compared interaction energy to the anticipated average binding affinity, it found that the lower the interaction energy, the lower the average binding affinity. Table 7 presents a summary of the findings. Hence, it was determined that peptides BR582 and BR599 displayed the highest potential to be developed into therapeutic drugs.

### 3.5. Analysis of Peptides BR582 and BR599 with The Omicron Variant S Protein

**Figure 12:**
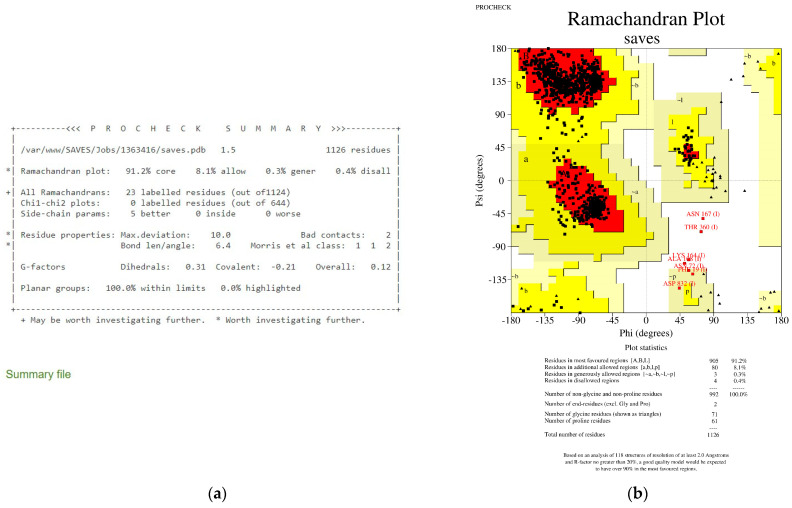
PROCHECK analysis for Omicron S protein shows 91.20% residues in the most favoured regions and an overall G-factor of 0.12. (**a**): PROCHECK summary. (**b**): Ramachandran Plot showing residues in the most favoured region (red); additional allowed regions (yellow); generously allowed regions (light yellow); and disallowed regions (white).

**Figure 13:**
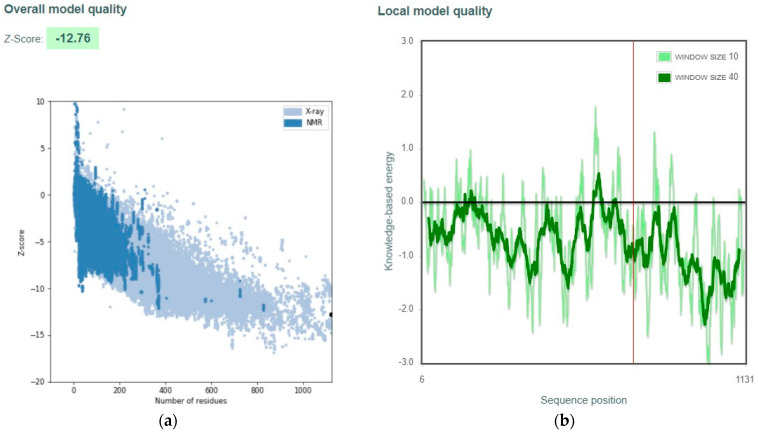
ProSA analysis for Omicron S protein chain A. (**a**): Z-score is −12.76. (**b**): Local model quality where most residues showed negative energy.

PROCHECK and ProSA confirmed that the homologically modelled Omicron S protein chain A was of excellent model quality and could be used for analysis with the two peptides. Figure 12 shows the PROCHECK summary of 91.2% of the modelled Omicron S protein chain A residues found in the most favoured regions with the overall G-factor at 0.12. These values described an excellent model. ProSA analysis, as shown in Figure 13, further supported that the Omicron S protein is of high-model quality.

**Figure 14:**
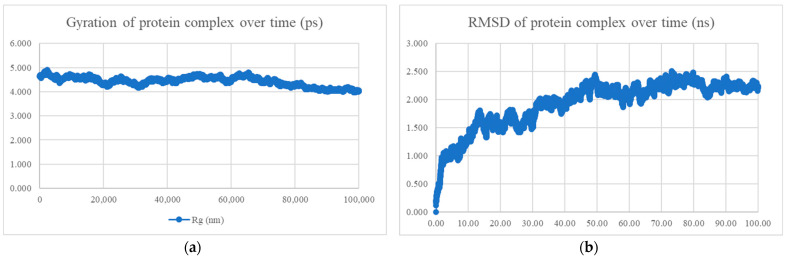
MDS analysis of the Omicron S protein and BR582 complex. (**a**) The gyration of the protein complex. A relatively constant line with an overall fluctuation of less than 1 nm shows that the protein complex is compact over time. (**b**) The RMSD of the protein complex. The RMSD initially increased due to minimization and reached stability around 2.25 nm, showing that the protein complex is stable over time. The protein complex’s interaction energy (IE) is −397.472 ± 20.1 kJ/mol, and the mean number of total hydrogen bonds between the protein complex is 4.90.

BR582 and BR599 were docked with the Omicron variant’s S protein, and the docked complexes were subjected to 100 ns MDS analysis. PCA analysis showed that the major motion of the two peptide-Omicron S protein complexes (more than 90%) lies in the first 2 and 3 PCs. The cosine content of both complexes was 0.54 and 0.72, respectively. Since the cosine content of the BR599-Omicron S protein complex was above 0.7, the MDS results were not analysed. Table 8 shows the docking result, PCA and cosine content analysis, and the MDS results for the peptide-Omicron S protein complexes. The stable Rg and RMSD values from MDS indicated that the BR582-Omicron S protein complex was compact and stable throughout 100 ns, as shown in Figure 14. However, the lower interaction energy, −397.472 ± 20.1 kJ/mol, and the lower mean number of hydrogen bonds, 4.90, of the complex compared with that of the BR582-wildtype S protein suggested that the BR582 peptide has weaker interaction with the Omicron S protein. 

This could be due to the D614G, H655Y, N679K, and P681H mutations near the S1/S2 cleavage site of the Omicron variant. It was noted that the mutations involved changes in the amino acid groups, for instance, D (acidic) to G (non-polar), H (basic) to Y (polar), N (polar) to K (basic), and P (non-polar) to H (basic). Since BR582 was initially designed to target the wildtype S protein, these changes might affect the interaction of the peptide with the Omicron S protein. Hence, peptides targeting the Omicron S protein need to be designed in a separate study. 

This in silico study has provided preliminary data in developing therapeutic peptides to counter COVID-19. While the in silico results were both promising and exciting, there was an underlying concern about the ability of the peptides to interact with the S protein S1/S2 cleavage site and carry out its inhibiting function. The main concern lies in the fact that the S protein is glycosylated, and the role of glycans on the S protein is not yet determined. However, there were reports on the effect of glycosylation as a shield masking epitopes of the S protein from the immune system and hindering antibody recognition [86,87]. There was also a report on the S309 antibody recognizing an epitope containing a glycan on the RBD of the S protein, suggesting that glycosylation on the S protein is important in producing neutralizing antibodies [88]. Watanabe et al. (2020) also stated that the S protein of SARS-CoV-2 is less densely glycosylated when compared to S proteins of MERS-CoV and SARSCoV and is hence more efficient in eliciting an immune response [89].

A review by Zhao et al. (2021), discussing the roles of glycans on the SARS-CoV-2 S protein, stated that S protein glycosylation seemed to improve S and ACE2 interaction [87]. Another review article by Gong et al. (2021), which provided an important overview of N-glycosylation and O-glycosylation of SARS-CoV-2, stated that the N-glycosylation at N61, N603 and N657 proximal to the S1/S2 cleavage site was able to increase the steric hindrance for cleavage, which was beneficial for SARS-CoV-2 entry [90]. Although various O-glycosylation at S673, T678, and S686 near the S1/S2 cleavage site was also identified, their influence on S protein cleavage remains to be investigated [87,90]. An experiment performed in a cell culture system by Zhang et al. (2021) showed that proline mutations mimicking those seen in the highly transmissible alpha (P681H) or delta (P681R) variants showed decreased O-glycosylation and increased furin cleavage of the S protein in cell culture. The Omicron variant also has the same P681H mutation, therefore, a decreased O-glycosylation and increased furin cleavage are expected [91]. 

Hence, while glycosylation can introduce additional complexity to the S protein surface, it does not necessarily prevent peptide binding, since peptide–protein interactions typically occur through specific molecular recognition between the peptide and its target protein. The binding affinity and specificity can depend on various factors, including the sequence and structure of the peptide, as well as the accessible regions on the protein surface [92]. While glycosylation can affect the S protein’s spatial arrangement and surface properties, some regions remain available for peptide binding [87,90,91]. 

Since it is unclear whether the glycans themselves can shield or sterically hinder certain regions of the protein surface, potentially affecting peptide accessibility or enhancing the binding, experimental characterization, such as structural studies or binding assays, would be necessary to confirm the binding affinity further. Future studies should focus on in vitro and in vivo analysis of the peptides’ binding affinity to the S protein, and their inhibiting property in cell culture systems and animal models.

## 4. Conclusions

Peptides targeting the S protein’s S1/S2 cleavage site were designed and analysed in this study using bioinformatics tools such as HADDOCK, PEP-FOLD3, and GROMACS. According to the MDS data, six protein–peptide complexes were compact and stable, and six peptides were identified to have stable binding to the S protein over time. When binding to the S protein S1/S2 cleavage site, peptides BR582 and BR599 showed the highest interaction energy compared with other peptides, showing the highest potential to be developed into peptide drugs. Further laboratory testing is required to establish these peptides’ binding ability and inhibiting properties.

## Figures and Tables

**Table 1:** Docking analysis of S1/S2 cleavage site with host proteases.

No.	Model	HADDOCK Score	Cluster/Position	Cluster Size	RMSD	Z-Score	i-RMSD	FCC	Average Binding Affinity ΔG (kcal/mol)
1	S protein and Furin	−107.1 +/− 7.2	10.0/1.0	6	0.8 +/− 0.5	−2.5	0.0–1.0	0.5–1.0	−12.5
2	S protein and Trypsin	−122.0 +/− 9.2	1.0/1.0	62	0.8 +/− 0.6	−1.1	0.0–1.5	0.3–1.0	−12.3
3	S protein and TMPRSS2	−91.7 +/− 9.9	3.0/1.0	18	1.4 +/− 1.1	−2.4	0.0–3.0	0.4–1.0	−10.1
4	S protein and Matriptase	−101.5 +/− 5.8	2.0/1.0	41	1.3 +/− 0.8	−1.3	0.0–2.0	0.3–1.0	−11.8
5	S protein and Cathepsin B	−109.0 +/− 7.8	1.0/1.0	122	0.8 +/− 0.5	−1.7	0.0–1.2	0.4–1.0	−13.3
6	S protein and Cathepsin L	−131.6 +/− 7.7	2.0/1.0	27	0.8 +/− 0.5	−1.6	0.0–2.0	0.4–1.0	−13.8

**Table 2:** Interacting residues of host proteases at the S1/S2 site of the S protein.

	Furin	Trypsin	TMPRSS2	Matriptase	Cathepsin B	Cathepsin L
660S	-	-	-	92F	-	141E
661Y	119D, 120G, 121E	22Y, 43K	301P	92F, 168Q	196M	141E, 145F
662Q	76R, 118L, 119D, 120G	22Y		91D, 92F	195M, 196M	141E, 144L
663T	76R, 82D, 83N, 118L, 119D	40H, 174Q	296H, 299E	45I, 91D, 94F	195M, 196M, 197G, 198G	144L
664Q	76R, 77Y, 82D	174Q	-	-	-	-
665T	80M, 82D	174Q	-	-	-	-
666N	80M	128G	-	-	-	-
668P	85H	126S, 128G, 173C, 196G, 197C	342K, 461W, 463S	23Q, 47D, 49G, 50F, 52Y	245E	65C, 66N
669R	186N, 219W, 256T, 257G	193W, 195D, 196G, 199Q	342K, 417D, 419L	23Q, 26I, 52Y	245E	22C, 23G, 63E, 65C, 66N, 94T, 95E
670R	148E, 149D, 189R	193W	340K	-	75Y, 245E	21Q, 23G
671A	85H	174Q	296H	45I, 47D	245E	23G
672R	44D, 45D, 85H, 118L, 144S, 145W, 146G	40H, 172S, 173C, 174Q, 175G, 176D, 177S, 191V, 192S, 193W, 194G	296H, 337Y, 339S, 438Q, 441S, 460S, 461W	26I, 42H, 43C, 45I, 47D, 52Y, 188Q	173A, 174F, 175S, 198G, 199H, 240C, 241G, 244S, 245E	19Q, 23G, 25C, 66N, 67G, 162D, 189W
673S	148E, 149D	40H, 193W	296H, 299E, 339S, 340K	188Q	75Y, 76P, 198G, 245E	19Q, 21Q, 22C, 23G, 189W
674V	146G, 147P, 148E	78R	296H, 299E, 300K, 339S	141Y, 188Q, 212W, 213G, 214D, 215G	27G, 29C, 30W, 73G, 74G, 198G, 199H, 200A	18N, 19Q, 20G, 188S, 189W, 193W
675A	147P, 148E	-	339S	141Y	74G, 75Y	20G
676S		-	299E	-	74G, 198G	-
677Q	118L, 121E	43K	299E, 301P	94F	196M, 197G, 198G	144L, 189W, 193W
680I	76R	-	-	-	-	-

**Table 3:** Docking analysis of peptides designed to target the S1/S2 cleavage site of the S protein.

No.	Peptide Sequence	Amino Acid Length	Models Observed	HADDOCK Score	Cluster/Position	Cluster Size	RMSD	Z-Score	i-RMSD	FCC	Average Binding Affinity ΔG (kcal/mol)	Average Kd (M) at 25.0 °C	ToxinPred
BR556	FDRLYQEFKDDYEDSEER	18	166/200 models are alpha-helix	−125.1 +/− 2.6	1.0/1.0	71	0.9 +/− 0.5	−2.7	0.0–2.0	0.5–1.0	−10.8	1.51 × 10^−8^	Non-toxin
BR563	FDRLRQEFKDDFSEEEFR	18	141/200 models are alpha-helix	−126.1 +/− 2.1	1.0/1.0	70	0.8 +/− 0.5	−2.5	0.0–1.1	0.4–1.0	−10.1	3.80 × 10^−8^	Non-toxin
BR573	FDRLRQEFKDDFYESEFR	18	All alpha helix	−127.4 +/− 2.7	1.0/1.0	38	0.8 +/− 0.5	−2.7	0.0–1.9	0.4–1.0	−10.4	3.30 × 10^−8^	Non-toxin
BR582	FDRLRQEFKDDYEDEEERKE	20	110/200 models are alpha-helix	−130.3 +/− 2.3	1.0/1.0	76	0.7 +/− 0.4	−2.2	0.0–3.8	0.4–1.0	−10.9	1.04 × 10^−8^	Non-toxin
BR599	EKFDRLYQEFKDDYEDSEER	20	156/200 models are alpha-helix	−122.2 +/− 2.3	1.0/1.0	47	0.7 +/− 0.5	−2.7	0.0–2.2	0.5–1.0	−11.0	9.38 × 10^−9^	Non-toxin
BR605	FDRLRQEFKDDFYEEELREK	20	All alpha helix	−131.6 +/− 2.7	1.0/1.0	62	0.9 +/− 0.5	−2.2	0.0–1.8	0.4–1.0	−10.4	2.49 × 10^−8^	Non-toxin
BR606	FDRLRQEFKDDFYEEELRKE	20	All alpha helix	−134.5 +/− 0.6	1.0/1.0	62	0.8 +/− 0.6	−2.3	0.0–1.6	0.4–1.0	−10.2	3.48 × 10^−8^	Non-toxin
BR610	FDRLRQEFKDDFYESEFRKE	20	All alpha helix	−130.4 +/− 4.6	1.0/1.0	47	0.4 +/− 0.3	−2.3	0.0–1.6	0.5–1.0	−10.0	4.80 × 10^−8^	Non-toxin
BR614	FDRLRQEFKDDFYEEEERKE	20	All alpha helix	−129.6 +/− 5.7	1.0/1.0	54	0.8 +/− 0.6	−1.7	0.0–2.4	0.3–1.0	−10.6	2.02 × 10^−8^	Non-toxin

**Table 4:** Interacting residues of the peptides at the S1/S2 site of the S protein.

	BR556	BR563	BR573	BR582	BR599	BR605	BR606	BR610	BR614
641E	-	8F (v)	-	-	-	-	-	-	-
643V	-	8F (v)	-	8F (v)	10F (v)	8F (v)	8F (v)	-	8F (v)
645N	8F (v)	-	-	-	10F (v)	-	-	-	-
646S	L4 (v)	-	-	-	6L (v)	-	-	-	-
647Y	1F (v), 4L (v), 8F (v)	4L (v), 7E (v), 8F (v)	1F (v), 4L (v), 8F (v)	1F (v), 4L (v), 8F (v)	3F (v), 6L (v), 10F (v)	4L (v), 7E (v), 8F (v)	4L (v), 7E (v), 8F (v)	4L (v), 8F (v)	1F (v), 4L (v)
648E	1F (H, v), 3R (H, v), 4L (v)	1F (v), 3R (H, v), 4L (v)	1F (v), 3R (H, v), 4L (v)	1F (H, v), 3R (H, v), 4L (v)	2K (H, v), 3F (v), 6L (v)	3R (H, v), 4L (v)	3R (H, v), 4L (v)	3R (v), 4L (v)	1F (v), 3R (H, v), 4L (v)
650D	1F (v)	1F (v)	-	1F (v)	3F (v)	1F (v)	1F (v)	-	1F (v)
660S	-	1F (v), 4L (v)	1F (v)	1F (v)	3F (v)	1F (v)	1F (v)	1F (v)	1F (v)
661Y	1F (v), 2D (H, v), 5Y (v)	1F (v), 2D (v), 5R (v),	1F (v), 2D (H, v), 5R (v)	1F (v), 5R (v)	7Y (v)	1F (v)	1F (v)	1F (v), 5R (v)	1F (v), 2D (H, v), 5R (v)
662Q	1F (v), 5Y (v), 8F (v)	1F (v), 4L (v), 8F (v)	1F (v), 5R (v), 8F (v)	1F (v), 5R (v), 8F (v)	7Y (v), 10F (v)	1F (v), 5R (v), 8F (v)	1F (v), 5R (v), 8F (v)	5R (v), 8F (v)	1F (v), 5R (v), 8F (v)
663T	9K (v), 12Y (v), 13E (H, v)	8F (v), 9K (H, v), 12F (v)	8F (v), 9K (H, v), 12F (v)	8F (v), 9K (v), 12Y (v), 13E (H, v)	11K (v), 14Y (v), 15E (H, v)	8F (v), 9K (H, v)	8F (v), 9K (H, v), 12F (v)	5R (H, v), 8F (v), 9K (v), 12F (v)	8F (v), 9K (H, v), 12F (v)
664Q	12Y (v)	8F (v)	12F (v)	12Y (v)	14Y (v)	8F (v), 12F (v)	12F (v)	12F (v)	12F (v)
665T	12Y (H, v), 16E (H, v)	12F (v), 16E (H, v),	12F (v), 16E (v)	12Y (H, v), 16E (v)	14Y (H, v), 18E (v)	12F (v), 16E (v)	12F (v)	12F (v)	12F (v)
666N	-	16E (v)	16E (v)	12Y (v), 16E (v)	18E (v)	16E (v), 19K (H, v)	16E (v), 19K (H, v)	16E (v), 19K (H, v)	16E (v)
667S	16E (H, v)	16E (H, v)	16E (H, v)	16E (H, v)	18E (H, v)	16E (v), 20E (H, v)	16E (v), 20E (H, v)	16E (v), 20E (H, v)	16E (v)
668P	16E (v)	12F (v), 16E (v), 17F (v)	13Y (v), 16E (v), 17F (v)	16E (v), 17E (v), 20E (v)	18E (v), 19E (v)	13Y (v), 16E (v), 17L (v), 20E (v)	13Y (v), 16E (v), 17L (v), 20E (v)	13Y (v), 16E (v), 17F (v), 19K (v), 20E (v)	13Y (v), 16E (v), 17E (v), 20E (v)
669R	-	17F (v)	13Y (v), 17F (v)	20E (H, v)	-	13Y (v), 17L (v), 20E (H, v)	20E (H, v)	13Y (v), 17F (v), 20E (H, v)	13Y (v), 17E (v), 20E (H, v)
670R	-	-	13Y (v)	-	-	13Y (v)	-	-	13Y (v)
671A	13E (v), 16E (v), E17 (v)	9K (H, v), 13S (v), 16E (v)	9K (H, v), 12F (v), 13Y (v), 16E (v)	13E (v), 16E (v), 17E (v)	14Y (v), 15E (v), 18E (v)	9K (H, v), 12F (v), 13Y (v)	9K (H, v), 13Y (v)	9K (v), 12F (v), 13Y (v)	9K (H, v), 12F (v), 13Y (v)
672R	10D (H, v), 13E (v), 14D (H, v), E17 (H, v)	9K (v), 10D (H, v), 13S (v), 14E (H, v), 17F (v)	9K (v), 10D (H, v), 13Y (v), 14E (H, v)	10D (H, v), 13E (H, v), 14D (H, v), 17E (H, v)	11K (v), 12D (H, v), 15E (H, v), 16D (v), 19E (H, v)	9K (H, v), 10D (H, v), 13Y (v), 14E (H, v)	9K (v), 10D (H, v), 13Y (v), 14E (H, v)	9K (v), 10D (H, v), 13Y (v), 14E (H, v)	9K (v), 10D (H, v), 13Y (v), 14E (H, v)
673S	9K (H, v), 13E (H, v)	9K (H, v)	9K (H, v)	13E (H, v)	-	9K (H, v)	9K (H, v)	9K (H, v)	9K (H, v)
674V	9K (v)	9K (v)	-	-	-	-	-	9K (v)	9K (v)
676S	-	9K (v)	9K (v)	-	-	9K (v)	9K (H, v)	9K (H, v)	9K (v)
677Q	5Y (v), 9K (v)	5R (H, v), 9K (v)	5R (H, v), 9K (v)	-	7Y (H, v)	5R (v), 9K (v)	5R (v), 9K (v)	5R (H, v)	5R (H, v), 9K (v)
680I	-	8F (v)	-	-	-	8F (v)	8F (v)	-	-
685S	-	-	-	-	2K (H, v)	-	-	-	-
687G	-	-	-	-	2K (H, v)	-	-	-	-

**Table 5:** Physicochemical and ADMET analyses of the peptides.

Peptides	BR556	BR563	BR573	BR582	BR599	BR605	BR606	BR610	BR614
Physicochemical analysis									
Properties									
Molecular weight (g/mol)	2384.45	2393.55	2427.61	2676.79	2641.74	2692.92	2692.92	2684.90	2708.88
Theoretical pI	4.07	4.44	4.59	4.32	4.21	4.56	4.56	4.72	4.44
Molecular formula	C_104_H_146_N_26_O_3_	C_106_H_153_N_29_O_35_	C_110_H_155_N_29_O_34_	C_114_H_170_N_32_O_43_	C_115_H_165_N_29_O_43_	C_120_H_178_N_32_O_39_	C_120_H_178_N_32_O_39_	C_121_H_174_N_32_O_38_	C_119_H_174_N_32_O_41_
Number of atoms	315	323	328	359	352	369	369	365	366
Instability Index	60.48	78.59	66.99	71.73	55.44	84.27	84.27	61.30	90.94
Half-life (in h)	1.1 h	1.1 h	1.1 h	1.1 h	1.0 h	1.1 h	1.1 h	1.1 h	1.1 h
Hydrophobicity (%)	31.06	35.73	35.94	26.52	33.84	36.61	36.95	37.45	31.95
GRAVY	−2.133	−1.733	−1.611	−2.585	−2.290	−1.905	−1.905	−1.820	−2.270
ADMET analysis									
Absorption									
Water solubility (log mol/L)	−2.892	−2.892	−2.892	−2.892	−2.892	−2.892	−2.892	−2.892	−2.892
Skin perm (log Kp)	−2.735	−2.735	−2.735	−2.735	−2.735	−2.735	−2.735	−2.735	−2.735
P-glycoprotein substrate	No	No	No	No	No	No	No	No	No
P-glycoprotein inhibitors	No	No	No	No	No	No	No	No	No
Distribution									
Fraction unbound (human) (Fu)	0.381	0.381	0.381	0.381	0.381	0.381	0.381	0.381	0.381
Blood–brain barrier (BBB) permeability (logBB)	−5.846	−5.865	−5.665	−7.073	−6.408	−6.495	−6.497	−6.213	−6.767
Central Nervous System (CNS) permeability (logPS)	−10.995	−10.913	−10.75	−12.389	−11.982	−11.384	−11.459	−11.668	−11.914
Metabolism									
Cytochrome P 2D6 substrate	No	No	No	No	No	No	No	No	No
Cytochrome P 3A4 substrate	Yes	Yes	Yes	Yes	Yes	Yes	Yes	Yes	Yes
Cyctochrome P inhibitors	No	No	No	No	No	No	No	No	No
Excretion									
Total clearance (log mL/min/kg)	−2.773	−2.863	−2.824	−3.332	−3.24	−3.527	−3.383	−3.023	−3.386
Toxicity									
AMES toxicity	No	No	No	No	No	No	No	No	No
Maximum Recommended Tolerated Dose (MRTD) human (log mg/kg/day)	0.438	0.438	0.438	0.438	0.438	0.438	0.438	0.438	0.438
Rat oral LD50 (mol/kg)	2.482	2.482	2.482	2.482	2.482	2.482	2.482	2.482	2.482
Hepatotoxicity	No	No	No	No	No	No	No	No	No
Skin sensitization	No	No	No	No	No	No	No	No	No

**Table 6:** Result for PCA and cosine content analyses on the S protein and peptide complexes.

	No. of Eigenvectors (PC)	Eigenvalues of First Few PCs (%)	Cosine Content
S protein and BR556	54	PC1: 56.15	0.44
		PC2: 21.06	
		PC3: 11.20	
		PC4: 4.07	
		% of eigenvalues in 4 PCs = 92.48	
S protein and BR563	54	PC1: 47.98	0.14
		PC2: 21.80	
		PC3: 11.43	
		PC4: 7.07	
		PC5: 3.05	
		% of eigenvalues in 5 PCs = 91.33	
S protein and BR573	54	PC1: 74.41	0.34
		PC2: 19.28	
		% of eigenvalues in 2 PCs = 93.69	
S protein and BR582	60	PC1: 76.66	0.57
		PC2: 14.76	
		% of eigenvalues in 2 PCs = 91.42	
S protein and BR599	60	PC1: 65.37	0.21
		PC2: 12.13	
		PC3: 9.37	
		PC4: 4.94	
		% of eigenvalues in 4 PCs = 91.81	
S protein and BR605	60	PC1: 92.21	0.49
		% of eigenvalues in 1 PCs = 92.21	
S protein and BR606	60	PC1: 89.61	0.72
		PC2: 6.45	
		% of eigenvalues in 2 PCs = 96.06	
S protein and BR610	60	PC1: 67.47	0.53
		PC2: 19.50	
		PC3: 6.41	
		% of eigenvalues in 3 PCs = 93.38	
S protein and BR614	60	PC1: 80.92	0.86
		PC2: 11.65	
		% of eigenvalues in 2 PCs = 92.57	

**Table 7:** Summary of MDS analysis of peptides designed to target the S protein’s S1/S2 cleavage site.

No.	Peptide Sequence	Amino Acid Length	Models Observed	Average Binding Affinity (kcal/mol)	Radius of Gyration and RMSD	Interaction Energy (kJ/mol)	Mean Number of Hydrogen Bonds
BR556	FDRLYQEFKDDYEDSEER	18	166/200 models are alpha-helix	−10.8	Compact and stable	−698.794 ± 22.5	10.78
BR563	FDRLRQEFKDDFSEEEFR	18	141/200 models are alpha-helix	−10.1	Compact and stable	−603.423 ± 44.9	7.78
BR573	FDRLRQEFKDDFYESEFR	18	All alpha helix	−10.4	Compact and stable	−630.395 ± 28.2	6.90
BR582	FDRLRQEFKDDYEDEEERKE	20	110/200 models are alpha-helix	−10.9	Compact and stable	−1004.769 ± 21.2	13.85
BR599	EKFDRLYQEFKDDYEDSEER	20	156/200 models are alpha-helix	−11.0	Compact and stable	−1040.334 ± 24.1	14.51
BR605	FDRLRQEFKDDFYEEELREK	20	All alpha helix	−10.4	Not compact, not stable	−809.339 ± 42.3	11.53
BR610	FDRLRQEFKDDFYESEFRKE	20	All alpha helix	−10.0	Compact and stable	−651.934 ± 37.8	7.28

**Table 8:** Docking results, PCA, cosine content analyses, and MDS results for the two peptide-Omicron S protein complexes.

		BR582-Omicron S Protein	BR5599-Omicron S Protein
Docking results	HADDOCK score	−97.4 +/− 4.8	−100.8 +/− 6.9
	Cluster/Position	3.0/1.0	2.0/1.0
	Cluster size	20	17
	RMSD	0.7 +/− 0.5	1.0 +/− 0.6
	Z-score	−2.0	−2.2
	i-RMSD	0.0–3.9	0.0–3.5
	FCC	0.5–1.0	0.5–1.0
	Average binding affinity ΔG (kcal/mol)	−10.7	−10.7
	Average Kd (M) at 25.0 °C	1.58 × 10^−8^	1.65 × 10^−8^
PCA and cosine content analysis	No. of Eigenvectors (PC)	60	60
	Eigenvalues of first few PCs (%)	PC1: 63.58 PC2: 19.51 PC3: 10.42 % of eigenvalues in 3 PCs = 93.51	PC1: 77.95 PC2: 16.21 % of eigenvalues in 2 PCs = 94.16
	Cosine content	0.54	0.72
MDS results	Radius of gyration and RMSD	Compact and stable	-
	Interaction energy (kJ/mol)	−397.472 ± 20.1	-
	Mean Number of Hydrogen Bonds	4.90	-

## Data Availability

Data is available upon request.
